# Maturogenesis of an Immature Dens Evaginatus Nonvital Premolar with an Apically Placed Bioceramic Material (EndoSequence Root Repair Material®): An Unexpected Finding

**DOI:** 10.1155/2018/6535480

**Published:** 2018-06-07

**Authors:** S. Nagarajan M. P. Sockalingam, Mohd Safwani Affan Alli Awang Talip, Ahmad Shuhud Irfani Zakaria

**Affiliations:** Centre for Family Oral Health, Faculty of Dentistry, The National University of Malaysia (UKM), Kuala Lumpur, Malaysia

## Abstract

Dens evaginatus is a dental developmental anomaly that arises due to the folding of the inner dental epithelium that leads to the formation of an additional cusp or tubercle on the occlusal surface of the affected tooth. This accessory tissue projection may carry with it a narrow and constricted pulp horn extension. Occasionally, the tubercle easily fractures, thus leading to microexposure of the pulp horn and eventual pulp necrosis. Often, the pulp necrosis occurs at a time the root development of the affected tooth is incomplete. Apexification with calcium hydroxide and mineral trioxide aggregates has been the mainstay of treatment options before root canal obturation in immature nonvital permanent teeth. Lately, regenerative endodontics (maturogenesis) is becoming one of the preferred treatment modalities to manage such teeth. The current case highlights the possibility of a bioceramic material (EndoSequence Root Repair Material, BC RRM-Fast Set Putty™, Brasseler, USA) which supposed to provide apical root closure (apexification) and could also induce continuation of root growth (maturogenesis).

## 1. Introduction

Dens evaginatus (DE) is a rare dental anomaly that is common in 1–4% people of Asian descent and mainly observed in the premolars. The mandibular premolars are five times most likely to have this developmental anomaly than the maxillary premolars [1]. The outward projection of the tooth may appear as tubercle on the occlusal surface and consists of a dentine core with outer enamel coverage. There are four anatomical variations to the DE tubercle types, namely, smooth, grooved, terraced and ridged, as classified by Lau in 1955 [2]. Oehlers et al. in 1967 reported that 70% of tubercles have pulp tissue extensions into them [3].

One of the major clinical issues related to DE is the possible traumatic occlusion of the adjacent erupted tooth in contact. The traumatic forces may cause either attrition or fracture of the tubercle, thus resulting in inevitable pulp exposure [2]. Pulp exposure often leads to signs and symptoms of pulpitis, pulp necrosis, and apical periodontitis. In some of the reported DE cases, apical periodontitis had progressed into facial cellulitis and osteomyelitis [4].

The current case describes the management of a nonvital immature mandibular right second premolar tooth with DE that developed pulp necrosis and symptomatic apical periodontitis. Although apexification was performed successfully with a newly available bioceramic root repair material (EndoSequence®, BC RRM-Fast Set Putty, Brasseler, USA), the use of this material also resulted in the unexpected continued root growth (maturogenesis).

## 2. Case Report

A healthy 11-year-old girl was presented to the National University of Malaysia (UKM) Paediatric Dental Clinic with a referral for further management of pulp necrosis of an immature lower right second premolar (tooth 45), secondary to the fractured tubercle of dens evaginatus. Two weeks earlier, she had treatment at a general dental clinic for pain related to tooth 45. Tooth 45 had spontaneous and lingering pain following cold and thermal stimuli. The tooth was diagnosed to have symptomatic irreversible pulpitis, and root canal therapy was initiated. The canal was accessed, and pulp extirpation performed before the placement of intracanal nonsetting calcium hydroxide by the general dental practitioner (GDP).

**Figure 1:**
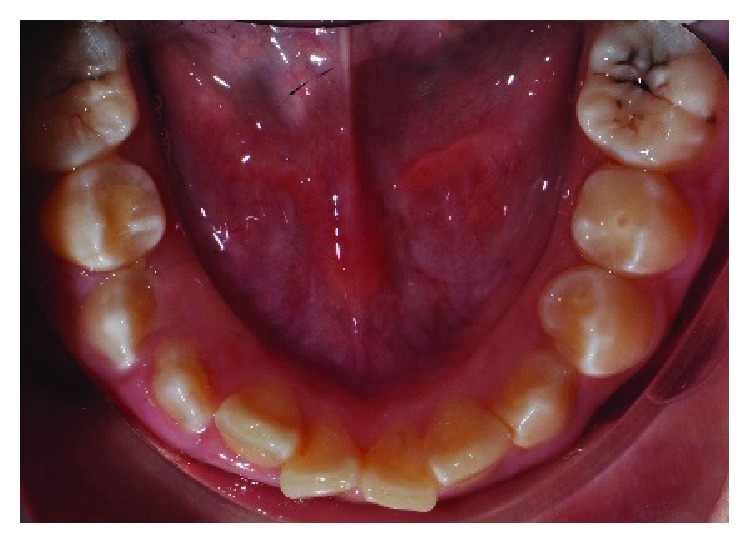
Clinical photograph of tooth 45 with an occlusal restoration of the access cavity and fractured dens evaginatus tubercle of tooth 35.

**Figure 2:**
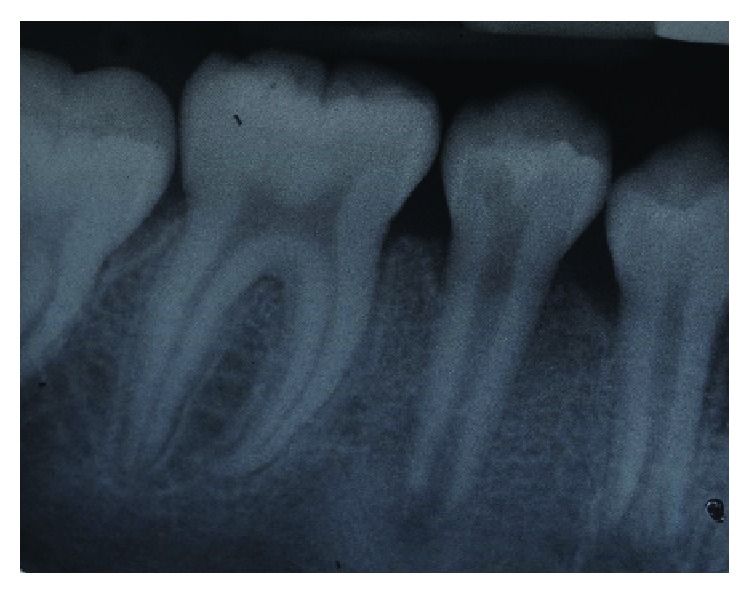
Periapical radiograph of tooth 45 showing immature root apex.

At the time of current assessment, her tooth-related symptoms had completely resolved. General oral examination showed the presence of generalised mild gingivitis with a basic periodontal examination (BPE) score of 1 in all sextants. The patient's oral hygiene was fair with a plaque score of 30%. The patient is still in her mixed dentition with the presence of the primary maxillary canines. Her upper dental arch was well aligned, and mild crowding of anterior teeth was noted in the lower arch. Tooth 45 has an occlusal glass ionomer dressing of the access cavity made for the pulp extirpation earlier by the GDP (Figure 1). Cold and electric pulp sensibility testings showed positive responses to all fully erupted premolars indicative of tooth vitality expect for tooth 45. Tooth 45 also has slight tenderness to percussion. Periapical radiograph of tooth 45 showed an immature root with convergent open apex and small periapical radiolucency. The pulp space of tooth 45 is of an even width from the coronal to the apical portion (Figure 2). Based on the assessments, tooth 45 was diagnosed with pulp necrosis secondary to fractured dens evaginatus and symptomatic apical periodontitis.

On the day of initial assessment, tooth 45 was isolated with rubber dam after infiltration of local anaesthetic solution (2% lidocaine with 1 : 80000 adrenaline). Pulp chamber was reentered through the previously prepared access cavity. The root canal was exposed and irrigated with saline. After that, the canal was dried with paper points and the tooth working length was estimated with a K-file No. 60. A working length, 2 mm short of the apical opening, was determined (17 mm). The canal was gently prepared with the K-file No. 60 and then irrigated with a copious volume of 1.5% sodium hypochlorite (NaOCI). After drying the wet canal with paper points, nonsetting calcium hydroxide was placed into the canal and the access cavity was double sealed with Cavit™ 3M, USA, and glass ionomer cement (GIC) (Riva Self Cure™ SDI, Australia).

**Figure 3:**
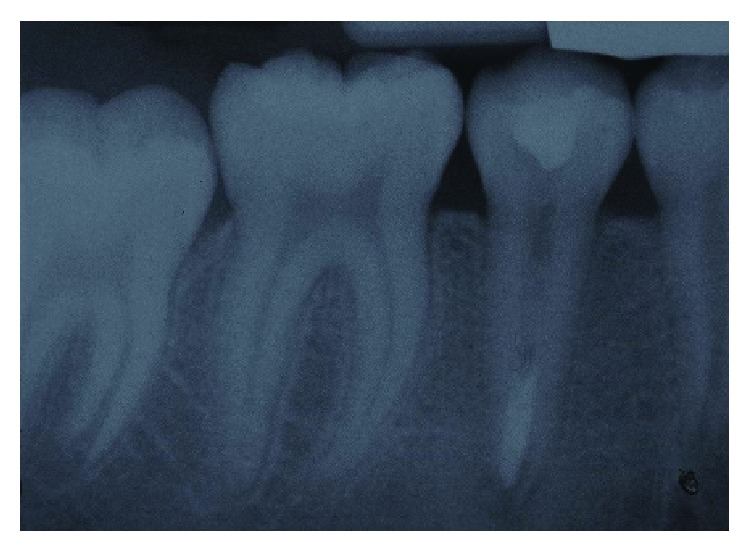
Periapical radiograph of tooth 45 after apexification with EndoSequence Root Repair Material.

Two weeks later, the tooth was reassessed for any signs and symptoms of infection. The tooth was no longer tender to percussion, and there was no indication of infection-related signs and symptoms. After isolation with a rubber dam, the root canal of tooth 45 was reaccessed and irrigated with a copious volume 1.5% NaOCl to remove the nonsetting calcium hydroxide. Then, the canal was irrigated with sterile water and dried with paper points. Subsequently, the canal was irrigated with 17% EDTA (Pulpdent™, Watertown, Massachusetts) for a minute and dried with paper points. Finally, under the guidance of a dental operating microscope (Carl Zeiss Surgical GmbH, S100), the apical region was filled using the EndoSequence (BC RRM-Fast Set Putty, Brasseler, USA) material up to 4 mm thickness to create an apical seal (Figure 3). The orifice of the root canal was double sealed with a cotton pellet, temporary filling material (Cavit 3M, USA), and GIC (Riva Self Cure SDI, Australia).

**Figure 4:**
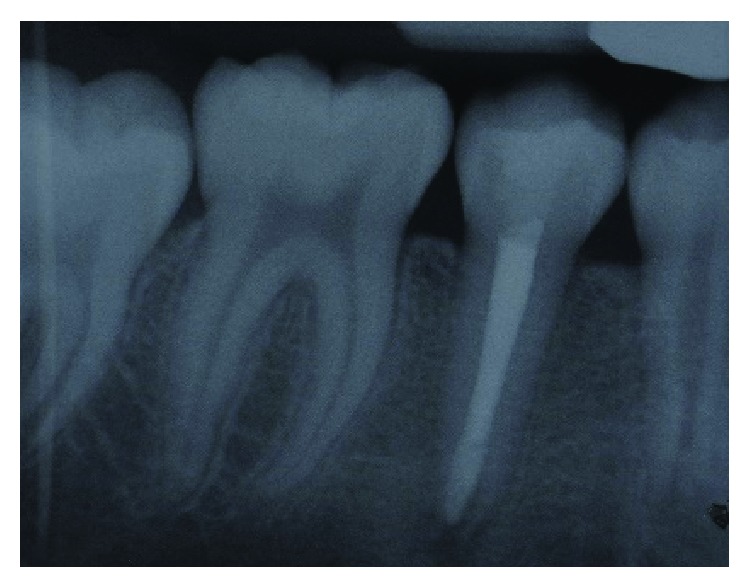
Periapical radiograph of tooth 45 after root canal obturation with thermoplasticised gutta-percha.

Once again, the root canal was reaccessed two weeks later, irrigated with 1.5% NaOCl, and dried with paper points. Next, the dried canal was obturated with thermoplasticised gutta-perca using the Obtura III Max System (Obtura Spartan® Endodontics) (Figure 4). After that, the access cavity was double sealed with GIC (Riva Self Cure SDI, Australia) and nanohybrid composite (AURA™ SDI, Australia), respectively.

**Figure 5:**
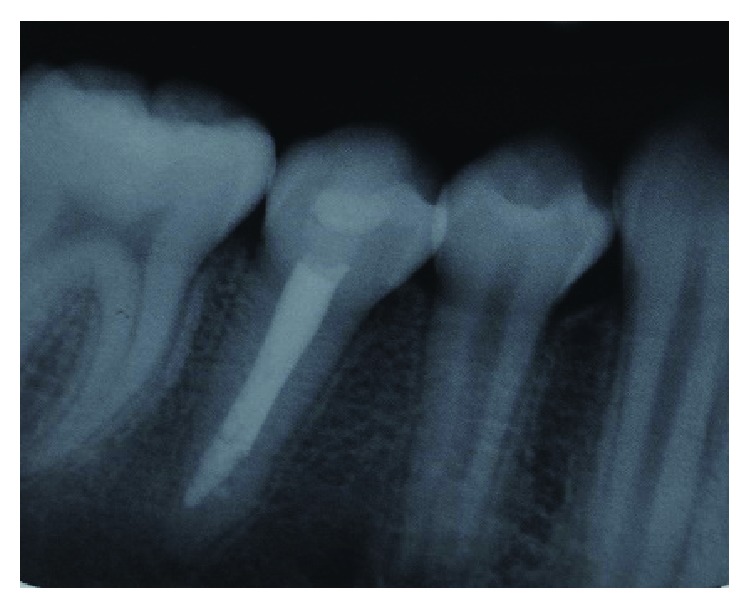
Periapical radiograph of tooth 45, 6-month postapexification showing complete root maturogenesis.

Following the obturation, tooth 45 was reviewed at three-month and six-month intervals. During both reviews, tooth 45 was asymptomatic. However, at the six-month review, a periapical radiograph of tooth 45 showed an unexpected finding. The apical root of tooth 45 continued to grow beyond the apexification level with a normal periodontal ligament space and lamina dura. No evidence of periapical radiolucency was noted (Figure 5). However, regular annual monitoring of tooth 45 is essential to ensure that the coronal seal is intact and no apical complication further arises.

## 3. Discussion

Numerous treatment modalities are available to treat teeth with dens evaginatus (DE) [2, 4, 5]. However, the treatment selection depends mainly on the stage of root development and symptomatic status of the affected tooth. Levitan and Himel in 2006 proposed a possible treatment protocol to manage teeth with DE based on whether the treatment is for teeth with healthy or diseased pulp. Dens evaginatus tooth with healthy pulp is treated either by reducing the occlusal contact of the opposing tooth or by strengthening its tubercle with a flowable composite resin regardless of whether the tooth has a mature or immature root. If the tooth has an inflamed pulp, partial pulpotomy with MTA is suggested for an immature tooth and conventional root canal treatment for the mature tooth. If the DE tooth has a necrotic pulp, apexification is proposed for an immature tooth and conventional root canal treatment for a mature tooth [2].

Necrotic teeth with immature roots provide significant challenges to clinicians especially with their wide open apices which do not allow successful canal obturation and thin dentine walls which are prone to fracture and compromised crown-root ratio [6]. Over the years, apexification with calcium hydroxide has been the mainstay of treatment for the immature teeth. Apexification allows apical barrier formation that enables condensation of root filling materials against it. However, the usage of calcium hydroxide for apexification had declined currently due to its shortcomings such as prolonged treatment time to achieve apical barrier, and it has hygroscopic and proteolytic properties that induce the desiccation of dentinal proteins [7–10]. In the last decade, mineral trioxide aggregates (MTA) have been used widely in the apexification of nonvital immature teeth. Although MTA has reduced the treatment time and had shown higher success rate than calcium hydroxide [11], nevertheless, it too has some setbacks such as tooth discolouration and weakening of the dentine wall due to the similarity in its effect to calcium hydroxide [10, 12, 13]. Of late, many other materials such calcium-enriched cement, bioaggregate, Biodentine, and EndoSequence Root Repair Material have been marketed for various endodontic procedures [14]. These materials showed some promising results, but the long-term evidence of their success is still scanty.

The regenerative endodontic technique (RET) is another treatment option that has received wide coverage in the literature regarding the management of nonvital permanent teeth in recent times. Although this technique is initially known as the vascularisation [15], other names for this technique are revitalisation, repopulation, regeneration, or even maturogenesis [6]. However, Wigler et al. in 2013 argued that this technique not only promotes blood vessel formation but also leads to continued root development; therefore, maturogenesis is a better term to use to describe the root growth [16]. This technique allows repopulation of the root canal with pluripotent cells from the apical papilla of the immature tooth and initiation of root development [15]. Maturogenesis is said to promote continued root development with increased dentine thickness, thus improving the long-term prognosis of the affected teeth [6]. This treatment technique has caused a paradigm shift in our management thoughts regarding nonvital immature teeth. Nevertheless, it is still premature to conclude that this method is successful in all cases.

In the described case, we performed an apexification procedure on necrotic immature tooth 45 after discussing the various treatment options with the child's parents. In this procedure, the EndoSequence Root Repair Material (ERRM) was used to create an apical barrier. This material is a premixed bioceramic material which is composed of calcium silicates, zirconium oxide, tantalum oxide, calcium phosphate monobasic, and filler agents [17]. The material has a putty-like consistency that allows easy placement through a syringe. It has high tissue compatibility, antibacterial action (pH > 12), and excellent sealing properties [18–21]. Besides these advantageous properties, the material also has both the osteoconductive and osteoinductive potentials. Hydration of the material leads to the release of calcium and hydroxide ions into the surroundings. Over time, with the presence of phosphate ions in the surroundings, osteoconduction occurs with hydroxyapatite crystal-like structures being deposited over the placed ERRM material, and this results in the apical barrier formation.

Though the intended treatment for this patient is to create an apical barrier for root canal obturation, an unexpected finding was observed during the follow-up stages after the root canal obturation. We found that the root continued to grow and achieved its matured development. A normal lamina dura with an even periodontal space was noted around the apex of the tooth. One of the reasons for this occurrence is probably due to the osteoinductive property of the ERRM. The osteoinductivity of ERRM could have attracted the pluripotent cells from the surroundings to form preosteoblasts. The presence of preosteoblasts leads to the secretion of the bone matrix that later calcified into bone [17]. Based on a literature search, this appears to be the first case that reports maturogenesis of the root of a necrotic nonvital immature permanent tooth using ERRM as an apexification material.

## 4. Conclusion

The current case showed that ERRM is not only successful in creating an apical barrier in a nonvital immature permanent tooth for root canal obturation but also able to promote continued root growth. However, the potential of ERRM in fostering continued root growth on nonvital immature permanent teeth needs further clinical and research evidence.
